# Polydopamine-Coated Cu-BTC Nanowires for Effective Magnetic Resonance Imaging and Photothermal Therapy

**DOI:** 10.3390/pharmaceutics15030822

**Published:** 2023-03-02

**Authors:** Senthilkumar Thirumurugan, Kayalvizhi Samuvel Muthiah, Rajalakshmi Sakthivel, Mei-Yi Liao, Hitoshi Kasai, Ren-Jei Chung

**Affiliations:** 1 Department of Chemical Engineering and Biotechnology, National Taipei University of Technology (Taipei Tech), Taipei 10608, Taiwan; 2 Department of Applied Chemistry, National Pingtung University, Pingtung 90003, Taiwan; 3 Institute of Multidisciplinary Research for Advanced Materials, Tohoku University, Sendai 980-8577, Japan

**Keywords:** photothermal therapy (PTT), polydopamine (PDA), magnetic resonance imaging (MRI), nanowires (NWs)

## Abstract

Herein, we present a one-pot hydrothermal approach for synthesizing metal–organic framework-derived copper (II) benzene-1,3,5-tricarboxylate (Cu-BTC) nanowires (NWs) using dopamine as the reducing agent and precursor for a polydopamine (PDA) surface coating formation. In addition, PDA can act as a PTT agent and enhance NIR absorption, producing photothermal effects on cancer cells. These NWs displayed a photothermal conversion efficiency of 13.32% after PDA coating and exhibited good photothermal stability. Moreover, NWs with a suitable T_1_ relaxivity coefficient (r_1_ = 3.01 mg^−1^ s^−1^) can be effectively used as magnetic resonance imaging (MRI) contrast agents. By increasing concentrations, cellular uptake studies showed a greater uptake of Cu-BTC@PDA NWs into cancer cells. Further, in vitro studies showed PDA-coated Cu-BTC NWs possess exceptional therapeutic performance by 808 nm laser irradiation, destroying 58% of cancer cells compared with the absence of laser irradiation. This promising performance is anticipated to advance the research and implementation of copper-based NWs as theranostic agents for cancer treatment.

## 1. Introduction

Hepatocellular carcinoma (HCC) is one of the deadliest cancers in the world, causing cancer-related deaths when distant neoplasms develop [1,2,3,4,5,6]. Most patients are diagnosed late stage, resulting in poor diagnosis and treatment. The detection of potentially deadly cancers such as HCC remains challenging. Surgical resection has been shown to prolong the lives of individuals with HCC or liver metastasis, especially colorectal cancer liver neoplasm. The final treatment used to treat HCC is regular chemotherapy, which causes side effects, reduces patient comfort, and causes patients to worsen. Due to the limitations and adverse effects of the previous approaches, it is desired to seek out alternatives, particularly those that enable focused therapy, in order to increase patient comfort and therapeutic efficacy. As a result, there are high hopes for nanotechnology, which is anticipated to boost the effectiveness of existing treatments and generate new alternative modalities, therefore minimizing or eliminating undesirable side effects and toxic effects to normal tissues [7]. Conventional chemotherapy is considered one of the most efficient cancer treatments; however, it has some adverse effects, including the destruction of healthy tissues. Consequently, it is essential to create a combined treatment process that incorporates several therapeutic treatments, such as chemotherapy–photodynamic therapy [8,9,10,11,12], photodynamic–photothermal therapy (PTT) [13,14,15,16,17,18], and chemotherapy [19,20,21,22,23].

PTT is one of the most promising cancer treatments, owing to its low invasiveness, reduced adverse effects, and specificity [24,25,26]. It typically uses near-infrared lasers to irradiate nanoparticles that reach the tumor site, where light is transformed into heat to eradicate tumor cells thermally. PTT uses photothermal transduction agents (PTAs) to generate sufficient heat energy via the PTT effect, thereby exacerbating tumor cell ablation. Absorption of PTAs was predominantly distributed between 750 and 1350 nm. Overall, the thermal effect can only be produced when NIR light is imposed on the site where the PTA is available. Thus, the PTT of cancer causes minimal damage to adjacent normal tissues [24]. Therefore, PTT is considered a promising non-invasive treatment technique. It has been established that PTT can successfully remove a variety of cancer types. The effectiveness of thermal treatment for cancer is predicated on the cancer cells’ sensitivity to heat. Although hyperpyrexia can cause irreversible damage to the membranes of cancer cells and induce protein denaturation, the tolerance of cancer cells to hyperpyrexia is weaker than that of healthy cells. This is due to the fact that cancer cells are more likely to die from the effects of hyperpyrexia. Under these conditions, thermal treatment has the potential to be an effective and selective therapeutic strategy. Moreover, the aggregation of exogenous PTAs in tumor cells is greater than that of the surrounding healthy tissue cells, which may further improve the efficacy of PTT. An ideal PTA would not overlap with the tumor background and have a high PCE. Additionally, it must preferentially accumulate in the tumors (target-specific). Thus, the creation of new PTAs is currently experiencing a “blooming” period of growth in the scientific community. As a result of the rapid growth of nanotechnology, numerous nanomaterials with different physical and chemical properties, including light-to-heat, are suited for PTT use. The EPR effect is very practical for accumulating photothermal nanoparticles in tumors. Nanomaterial-based PTAs are able to achieve a greater PCE than simple PTAs, and they have the ability to be combined with different imaging and diverse therapeutic functions for more effective cancer treatment. In the case of laser irradiation as a treatment for early cancer, the heat produced by the external laser equipment might affect the healthy tissue around the lesion region while it is being burned. Therefore, the clinical use is restricted due to the fact that this adverse effect is not selective. For this reason, the study and development of a photothermal effect that only results in local heating may have significant potential application benefits [27]. Furthermore, it has been found that the efficacy of PTT depends on photothermal agents. Investigations are currently being conducted to develop multifunctional theranostic PTAs that integrate with imaging modalities and photothermal abilities. These PTAs will serve as a platform for simultaneous PTT, diagnosis, and monitoring, which will increase the therapeutic efficacy of photothermal therapy (PTT) and reduce the risk of unwanted side effects [28].

Polydopamine (PDA) is a polymer derived from a mussel that was described for the first time in 2007. Since then, it has been utilized extensively in a variety of subfields within material chemistry as well as nanomedicine. There are a number of distinctive characteristics that contribute to the practical usage of PDAs in the production of interesting materials. Firstly, PDA possesses robust adhesive characteristics, which make it possible to coat nearly any kind of material. This includes hydrophobic surfaces, noble metals, and various groups of nanoparticles. Secondly, it has been demonstrated that PDA coating is biocompatible. Because of this, numerous research groups have taken an interest in PDA as a potentially helpful material for the fabrication of multifunctional nanomaterials for use in cancer therapy. PDA has also seen a significant amount of use in this field, particularly over the course of the past five years [7]. PDA has emerged as a promising PTT agent owing to its excellent physiological stability, photothermal conversion capability, biocompatibility, and degradability [29,30,31,32]. Furthermore, PDA can be obtained conventionally by dopamine monomer-derived spontaneous oxidation and self-polymerization at pH 8.5 and room temperature [32]. PDA can be functionalized with various nanomaterials due to its excellent adhesion properties, significantly enhancing cancer therapeutic effectiveness [30,31,32].

Owing to their porous structure, excellent biocompatibility, and high stability in extreme environments, metal–organic frameworks (MOFs) have attracted the attention of numerous researchers over the past decade [33]. MOFs are constructed from metallic and organic linkers and appear to have porous crystalline systems with an increased porous volume, biodegradability, surface area, and flexible functionalities that have a promising future in biological applications, including delivery systems and imaging [34,35]. Zeolitic imidazolate framework-8 is a nontoxic MOF composed of zinc ions that operate as nodes linked by 2-methylimidazolate linkages [36]. Because of the enhanced permeability and retention (EPR) effect, these MOFs might accumulate efficiently in tumor tissues [37]. Because of their outstanding NIR absorption and hyperthermic response under NIR irradiation, copper-based nanomaterials have recently attracted a great deal of attention in biomedical applications. In addition, copper nanoparticles (Cu NPs) are inexpensive, biocompatible, simple to degrade, and they are also simple to produce. As a result, copper was rapidly investigated as a potential agent in the field of PTT. Photodynamic therapy is an additional form of non-invasive phototherapy that has regulatory approval for the treatment of a variety of disorders, including psoriasis, age-related macular degeneration, and some oncological diseases [38].

This paper proposes a one-pot hydrothermal method for synthesizing NWs that effectively ablate HCC using PTT therapy. The conjugation of PDA resulted in the synthesis of NWs with superior PTT conversion efficiency and improved thermal stability. In this study:

- We developed Cu-BTC@PDA NWs for effective PTT treatment of HCC.
- As-prepared NWs serve as an outstanding T_1_ MRI contrast agent.
- It was found that the PTT conversion efficiency of NWs was 13.32%.
- As a result of their high conversion efficiency, NWs are able to eliminate HCC in the presence of laser light.

The rest of the manuscript is organized as follows: Section 2 discusses the materials and methods used to prepare Cu-BTC NWs, as well as their PTT characteristics, MRI, and cytotoxicity against cells. Section 3 covers our proposed results for the characterization of Cu-BTC@PDA NWs, heat generation of NWs at varying concentrations, contrast agent, and in vitro PTT therapy. Section 4 contains a discussion of the outcomes. Section 5 summarizes the work and discusses potential future research pertaining to our study.

## 2. Materials and Methods

In Section 2, the preparation and conjugation of PDA with NWs, evaluation of PTT properties, MRI analysis, and in vitro PTT effect on HCC are described.

### 2.1. Chemicals and Reagents

Zinc nitrate hexahydrate (Zn(NO_3_)_2_·6H_2_O), copper nitrate trihydrate (Cu(NO_3_)_2_·3H_2_O), 2-methylimidazole (2-MIM), trimesic acid (TMA), Glacial acetic acid, ethyl alcohol, Dopamine, Tris buffer solution, and triethylamine (TEA) were purchased from Showa Chemical Industry (Tokyo, Japan) and Showa Chemical Industry (Austin, TX, USA).

### 2.2. Characterization

The crystallinity of the Cu-BTC composite was investigated using X-ray powder diffraction (XRD; X’Pert3 Powder, Malvern PANalytical, Malvern, UK). Using a field emission scanning electron microscope (FESEM; JSM-7610F, JEOL Ltd., Akishima, Japan) equipped with an energy-dispersive X-ray analysis (EDX) system, the morphological characteristics and elemental composition of Cu-BTC were evaluated. In addition, the functionality and qualitative and chemical compositions of the materials were evaluated using Fourier-transform infrared (FTIR) spectroscopy (Spotlight 200i Sp2 with AutoATR System, Perkin Elmer, Waltham, MA, USA) and X-ray photoelectron spectroscopy (XPS; JPS-9030, JEOL Ltd., Minato-ku, Japan).

### 2.3. Preparation of Cu-BTC NWs

First, 0.2415 g of Cu(NO_3_)_2_·3H_2_O was supplemented with a mixture of TEA (0.418 mL), glacial acetic acid (0.62 mL), and ethyl alcohol (12 mL) in a beaker, which was agitated for 1 h at ambient temperature. Subsequently, 0.21 g of TMA was added to the solution and constantly stirred for 2 h. After transferring the mixture to an autoclave made of stainless steel, a hydrothermal reaction was conducted for 24 h at 85 °C. Finally, a blue precipitate was obtained. It was then washed with ethanol three times and allowed to dry for 12 h at 60 °C.

### 2.4. Preparation of PDA-Coated Cu-BTC NWs

To coat the Cu-BTC with a PDA shell, 1 mL of Cu-BTC solution (1 mg/mL) was mixed with 10 mL of 10 mM Tris buffer solution containing dopamine (pH 8.5) and sonicated continuously for 1 h at ambient temperature. Later, crude PDA-coated Cu-BTC (Cu-BTC@PDA) was purified by centrifugation at 10,000 rpm for 10 min. Finally, the obtained Cu-BTC@PDA NWs were redistributed in Nanopure water.

### 2.5. Photothermal Effect of Cu-BTC@PDA

First, the photothermal effects of Cu-BTC@PDA NWs in an aqueous solution were investigated. Various concentrations of Cu-BTC@PDA NWs (0.1, 0.2, 0.5, 1.0 and 2.0 mg/mL) were illuminated with 808 nm laser irradiation (1 W/cm^2^) for 300 s. Every 30 s, the temperature of the sample was monitored using a thermal infrared camera.

### 2.6. PTT Conversion Efficiency

To determine the photothermal conversion efficiency (η), the difference in temperature versus time was monitored under laser irradiation (808 nm) at 1 W/cm^2^ until the solution reached a stable state. After closing the irradiation source, the rate at which heat was transferred from the solution to the surrounding environment was determined by measuring the drop in temperature of the aqueous solution. The value was determined using the following Equation [39]:

(1) $$\eta =\frac{hS\;\left(Tmax-Tsurr\right)-Q_{dis}}{I\left(1-10^{-A_{808}}\right)}$$

where *h* and *S* are the heat transfer coefficient and surface area of the container, *Tmax* is the maximum temperature, and *Tsurr* is the temperature of the environment. *Q_dis_* is the heat accompanied by the absorbance of light, which was individually calculated using a cuvette cell comprising purified water in the absence of prepared materials. *I* is the power density of the laser (1 W/cm^2^) and *A*_808_ is the absorbance of Cu-BTC@PDA at 808 nm (*A*_808_ = 0.1804). The *hS* value was calculated by the following Equation [36]:

(2) $$Ꞇs=\frac{\mathrm{mD}\;\mathrm{CD}}{hS}$$

where Ꞇs is the constant time of the sample, mD and CD are the mass and heat capacity of purified water, respectively.

### 2.7. In Vitro MRI

In vitro MRI of Cu-BTC@PDA NWs was detected on a 7T MRI Bruker apparatus. Various concentrations of Cu-BTC@PDA NWs (0, 31.25, 62.5, 125, 250, 500, and 1000 µg/mL) were dispersed in agarose gel. After that, the longitudinal relaxivity value (r_1_) was attained by curve fitting, merging the 1/T_1_ relaxation time with numerous Cu concentrations.

### 2.8. Cellular Uptake of NWs

#### 2.8.1. Preparation of Fluorescently Labelled Cu-BTC@PDA NWs

The resultant nanocarrier was fluorescently labelled with FITC. This is accomplished by progressively adding a methanolic solution of FITC to the proper concentrations of Cu-BTC@PDA NWs in PBS (pH 7.4). This reaction was conducted at ambient temperature for 24 h and shaken in the dark. After 24 h, FITC-conjugated Cu-BTC@PDA NWs were washed and dried.

#### 2.8.2. Fluorescence Microscopy (FM) Analysis

As mentioned earlier, the HepG-2 cell line was cultivated at a concentration of 4 × 10^5^ cells on coverslips in 35 mm culture dishes. The cells were subsequently treated with FITC labelled Cu-BTC@PDA NWs (100 µg mL^−1^) under identical conditions. After 4 h, the cells were washed and fixed with 4.0% formaldehyde for 5 min at room temperature. Because 4′-6-diamidino-2-phenylindole (DAPI) binds to DNA and serves as a nuclei marker, it was employed to visualize the nuclei of cells. The cells were then rinsed and stained with a DAPI/PBS solution for 5 min. Finally, fluorescence microscopy was employed to examine the cells. DAPI excitation and emission wavelengths were 364 and 461 nm, while FITC were 481 and 515 nm.

### 2.9. Cell Viability and PTT Effect Assay

HepG-2 cells were used in the 3-(4,5-dimethyl-thiazol-2-yl)-2,5-diphenyltetrazolium bromide (MTT) assay to evaluate the in vitro cytotoxicity of Cu-BTC@PDA. Cells (2 × 10^4^) were seeded in a 96-well plate and incubated for 24 h. In addition, different concentrations of Cu-BTC@PDA NWs were supplemented to HepG-2 cells in the presence and absence of laser irradiation (1 W/cm^2^) for 5 min. Following an additional 24 h incubation, the MTT was performed to quantify relative cell viability.

### 2.10. Intracellular Detection of ROS

To adhere HepG2 cells, 1 × 10^5^ cells per well were seeded in 12-well plates with complete DMEM for 8 h at 37 °C. Following a 4-h incubation with 100 μg/mL of Cu-BTC@PDA NWs, cells were stained for 30 min with 10 μM DCFH-DA. Subsequently, cells were washed multiple times with PBS before being visualized using inverted fluorescence microscopy.

### 2.11. Statistical Analysis

Statistical data were analyzed using the Statistical Package for Social Sciences (SPSS 18.0 for Windows, SPSS Inc., Chicago, IL, USA), and one-way analysis of variance (ANOVA) was used to identify statistically different datasets. Significant datasets with *p*-value < 0.05 are represented with * and those with *p*-values <0.01, <0.001, and <0.0001 are represented with **, ***, or ****, respectively.

## 3. Results

### 3.1. Characterization of Cu-BTC@PDA NWs

**Scheme 1:**
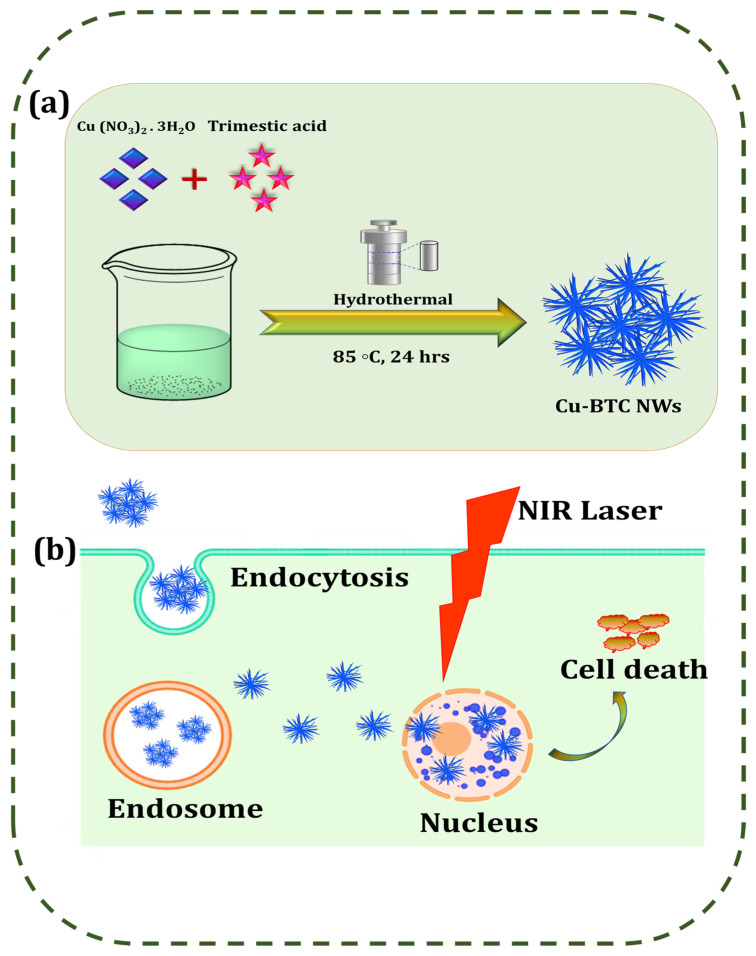
Schematic illustration for the (**a**) synthesis of Cu-BTC NWs and (**b**) internalized mechanism of Cu-BTC NWs.

**Figure 1:**
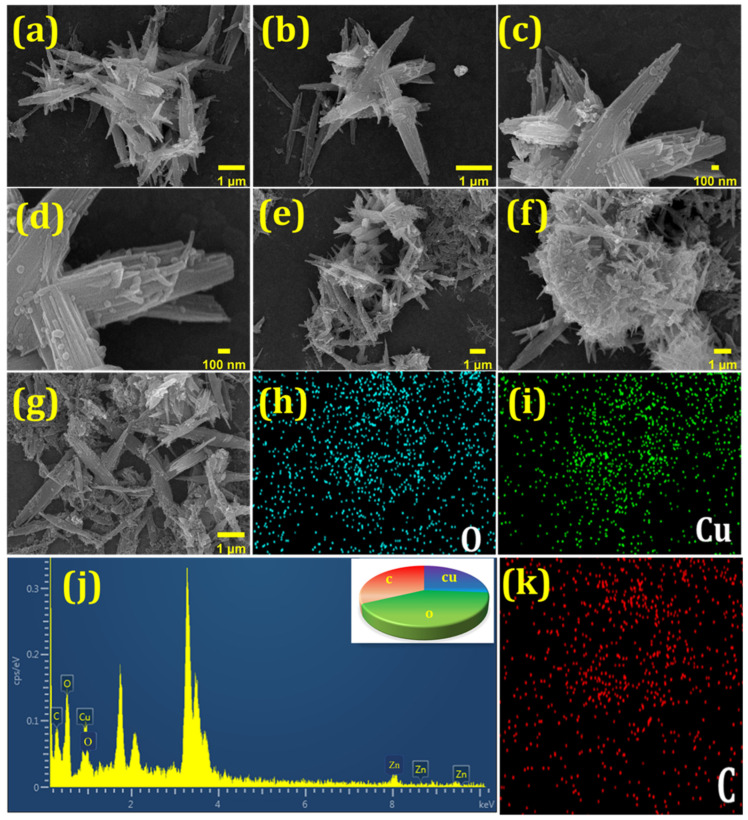
(**a**–**g**) FE-SEM images of Cu-BTC NWs. Mapping of (**h**) oxygen, (**i**) copper, and (**k**) carbon. (**j**) EDX analysis of Cu-BTC NWs.

Cu-BTC@PDA NWs were successfully synthesized using a one-pot hydrothermal method, as illustrated in Scheme 1. The surface morphology of Cu-BTC was examined by FESEM, which shows the formation of 1D nanowires (Figure 1a). EDX elemental mapping of copper (II) benzene-1,3,5-tricarboxylate (Cu-BTC) NWs is shown in Figure 1h–k. The EDX analysis depicts the presence of elements, and their weight percentages of copper (26.2%), carbon (32.1%), and oxygen (41.7%). These findings suggest the successful formation of Cu-BTC NWs.

**Figure 2:**
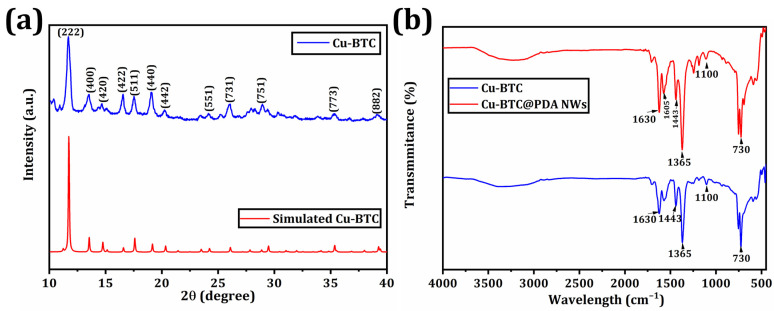
(**a**) XRD analysis of NWs. (**b**) FTIR analysis of NWs.

XRD was used to identify the crystalline nature of the prepared NWs, as shown in Figure 2a. The peaks at 11.52, 13.29, 14.82, 16.38, 17.34, 18.95, 20.08, 24.01, 25.83, 29.18, 35.04, and 39.02° were attributed to the (222), (400), (420), (422), (511), (440), (442), (551), (731), (751), (773), and (882) planes, respectively [39,40]. The FTIR spectrum of Cu-BTC is shown in Figure 2b. The peaks at 1365, 1443, and 1630 cm^−1^ are attributed to the carboxylic group of the trimesic ligand, stretching vibrations of the C=O bonds, and stretching mode of the C-O bond, respectively [41]. The weak absorption at 1100 cm^−1^ is ascribed to the symmetric and asymmetric stretching modes of O−C=O, and the strong absorption peak at 730 cm^−1^ is ascribed to the stretching modes of Cu−O [39]. Furthermore, FTIR was used to confirm PDA formation on the Cu-BTC NWs. The absorption peak at approximately 1605 cm^−1^ is assigned to the benzene skeleton vibration of PDA, which confirms the successful modification of PDA on Cu-BTC NWs [42].

### 3.2. XPS Analysis of Cu-BTC@PDA NWs

**Figure 3:**
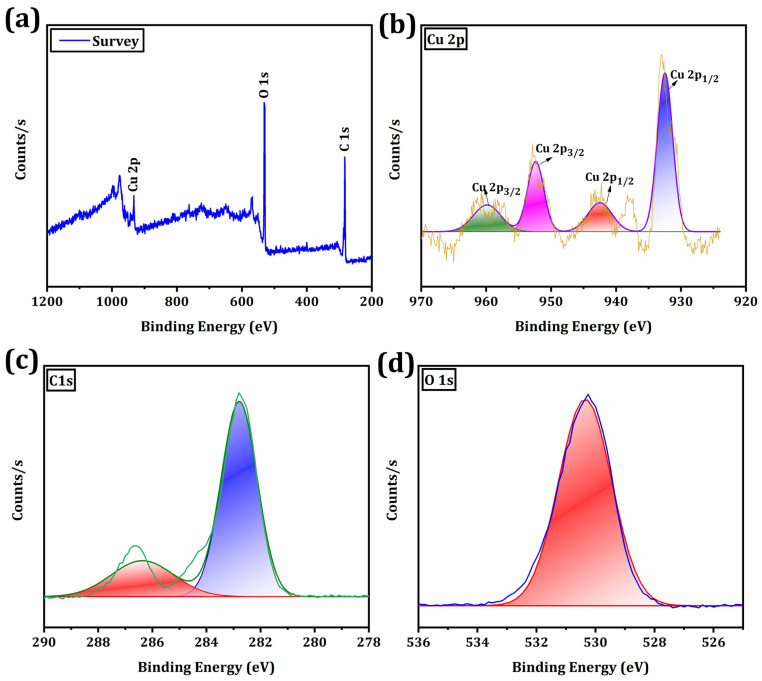
(**a**) Survey spectrum of NWs. (**b**–**d**) Core-level scan of copper (C 2p), carbon (C 1s), and oxygen (O 1s).

XPS analysis was used to identify the valence states of the as-prepared materials. As presented in Figure 3a, the peaks at 934, 531.8, and 285 eV in the XPS survey scan are attributed to Cu 2p, O1s, and C1s, respectively. The structure of the Cu^2+^ satellite is shown in Figure 3b. The peaks exhibiting binding energies of 932.52, 942.46, 952.42, and 960.01 eV are assigned to Cu 2p^1/2^, satellite Cu 2p^1/2^, Cu 2p^3/2^, and satellite Cu 2p^3/2^, respectively. Therefore, the valence state of the Cu-BTC@PDA NWs is most likely between +2. The XPS spectra of C1s are shown in Figure 3c. Evidently, the peaks are ascribed to phenyl and carboxyl signals, respectively. The O1s peak at 530.42 eV is attributed to Cu-O-C species, as shown in Figure 3d. The previously described findings suggest the successful formation of PDA on Cu-BTC NWs [43,44].

### 3.3. Photothermal Therapy

**Figure 4:**
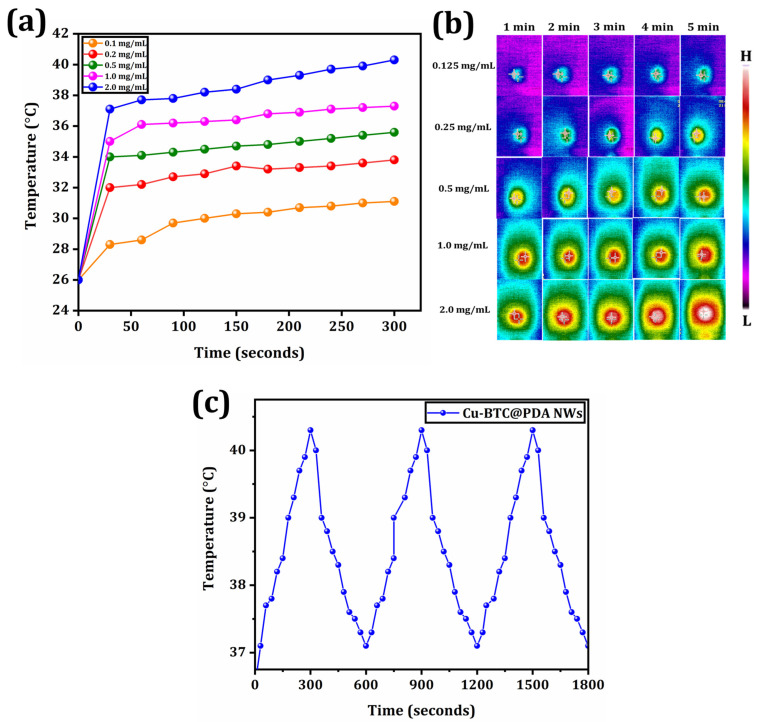
(**a**) Temperature elevation of Cu-BTC@PDA NWs. (**b**) IR Thermal images. (**c**) Photothermal stability (808 nm/1W/cm^2^).

The photothermal properties of the Cu-BTC@PDA NWs were investigated under 808 nm laser irradiation, as shown in Figure 4a. The temperature of the solution (2 mg/mL) was increased to 40.3 °C for 300 s, which is sufficient to kill cancer cells. This increase in temperature is because of the presence of the PTT contrast agent PDA. Under increasing temperature, the cells likely undergo a series of mild damages, including enzyme inactivation and mitochondrial injury, which leads to inducing cell death [45]. This suggests that the PTT properties of the Cu-BTC@PDA NWs are concentration-dependent. Figure 4b shows NIR images of different concentrations of Cu-BTC@PDA NWs. The color of the solution changed for different concentrations at varying time intervals owing to the NIR irradiation. Figure 4c depicts the heating and cooling curves of the Cu-BTC@PDA NWs under the ON and OFF lasers. The temperature of the solution remains the same for the Cu-BTC@PDA NWs under three consecutive cycles, indicating good photostability of the NWs. The time constant for heat transfer of the system is determined to be Ꞇs = 815.88 and the PTT conversion efficiency of the NWs is 13.32%. These findings suggest that Cu-BTC@PDA NWs are a promising candidate for PTT treatment of cancer.

### 3.4. MRI

**Figure 5:**
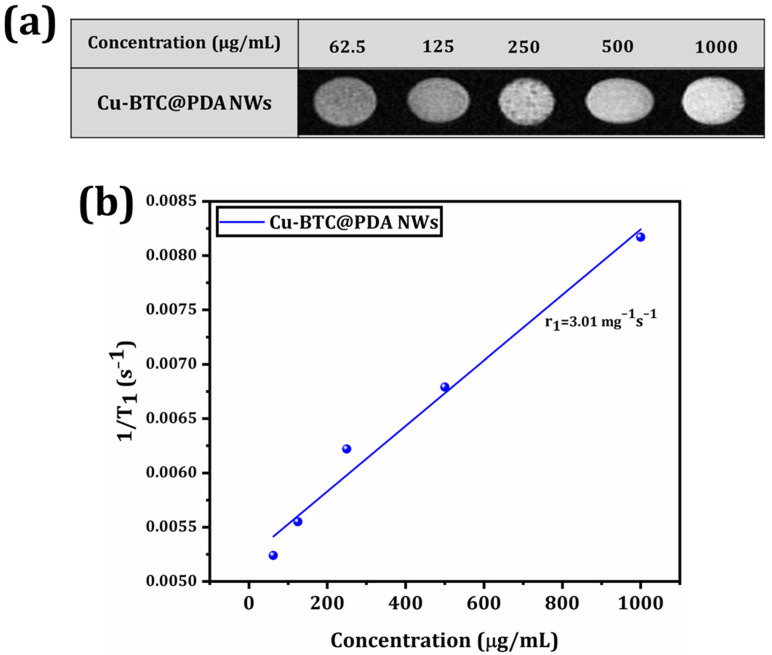
(**a**) In vitro T_1_ weighted MRI images of Cu-BTC@PDA NWs. (**b**) 1/T_1_ signal intensity of Cu-BTC@PDA NWs.

Owing to the paramagnetic properties of Cu^2+^, Cu-BTC nanowires are promising contrast agents for MRI. The T_1_-weighted MRI capacity of the Cu-BTC NWs was examined by analyzing Cu-BTC NWs in agarose gel suspensions of varying concentrations. Figure 5a illustrates the increasing signal intensities in T_1_-weighted MRIs obtained on an MR scanner, as demonstrated by the increased brightness in the Cu concentration range of 62.5 to 1000 μg/mL. The Cu concentration enhances the reciprocal transformation of T_1_ relaxation time, as depicted in Figure 5b. The evaluated fitting curve exhibited a linear characteristic with increasing Cu content. The T_1_ r_1_ is found to be 3.01 mg^−1^ s^−1^. These findings suggest that Cu-BTC@PDA NWs can act as potential contrast agents for MRI.

### 3.5. Cytotoxicity

**Figure 6:**
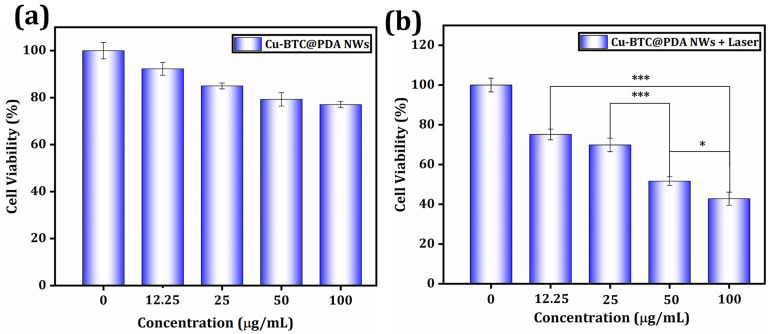
(**a**) Cytotoxicity and (**b**) photothermal therapy of HepG-2 cells with various concentrations of Cu-BTC@PDA NWs (808 nm/1 W/cm^2^). * *p* < 0.01 and *** *p* < 0.0001.

Cytotoxicity measurements were performed without laser irradiation to assess the biocompatibility of the as-prepared nanowires. As shown in Figure 6a, the cytotoxicity of the Cu-BTC@PDA NWs in the absence of laser irradiation exhibits excellent biocompatibility with liver cancer cells. This excellent biocompatibility demonstrates that using Cu-BTC@PDA NWs is safe for cancer therapy. However, in the presence of irradiation, the Cu-BTC@PDA NWs display a reduced viability of 68% at a concentration of 100 µg/mL, as shown in Figure 6b. This reduced viability is because the Cu-BTC@PDA NWs efficiently convert light into heat under irradiation, which helps kill cells. Altogether, these findings reveal that Cu-BTC@PDA NWs have a superior capability to kill cancerous cells under laser irradiation.

### 3.6. Detection of ROS

**Figure 7:**
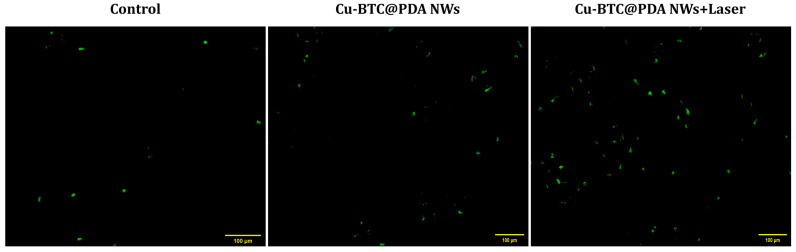
Fluorescence images of DCFH-DA-stained HepG2 cells treated with Cu-BTC@PDA NWs before and after laser (1 W/cm^2^) irradiation (scale bar: 100 μm). Green color indicates intracellular ROS.

The intracellular ROS can convert non-fluorescent DCFH to fluorescent DCF, which displays green fluorescence [46,47,48,49]. Similarly, the DCF (green) fluorescence signal was minimal in the control and Cu-BTC@PDA NWs group fluorescence images (Figure 7) prior to PTT treatment. The Cu-BTC@PDA NWs group exhibited a bright, large-scale green fluorescence following laser irradiation. Due to their PDA coatings, Cu-BTC@PDA NWs successfully eliminated ROS produced during PTT, as suggested by these results. These data confirmed that the cell death reported in Figure 6 was a result of high temperature.

### 3.7. Cellular Uptake of NWs

**Figure 8:**
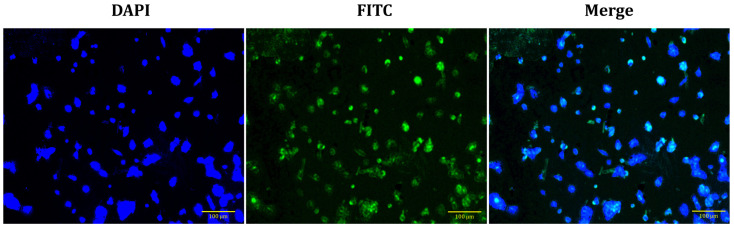
Cellular uptake analysis of FITC labelled Cu-BTC@PDA NWs in HepG-2 cells.

Cu-BTC@PDA NWs/FITC was added to the labelled cells at a concentration of 100 µg mL^−1^. Figure 8 depicts the accumulation of green and blue fluorescence caused by Cu-BTC@PDA NWs/FITC and DAPI nuclear staining. In addition, HepG-2 cells took up Cu-BTC@PDA NWs/FITC, which were primarily localized in the cytoplasm. The merged images of DAPI and FITC signals confirmed the uptake of Cu-BTC@PDA NWs/FITC by HepG-2 cells during incubation.

## 4. Discussions

We present a unique viewpoint regarding the conjugation of PDA with Cu-BTC NWs. PDAs have excellent NIR absorption rates, indicating they have great promise for PTT applications. Within 300 s, the PDA-conjugated Cu-BTC NWs (2.0 mg/mL) exhibited an increase in temperature. Under 808nm laser irradiation, Cu-BTC@PDA NWs demonstrated high PTT conversion capability and better thermal stability during three consecutive cycles. As a result of their paramagnetic characteristics (Cu^2+^, 3d orbitals: 3d^9^), NWs act as a T_1_ MRI contrast agent [50].

In addition, the NWs displayed ROS, as confirmed by DCFH-DA staining. Thus, confocal microscopy was utilized to visually demonstrate the internalization of Cu-BTC@PDA NWs by cells, confirming the presence and efficiency of Cu-BTC@PDA NWs’ incorporation in the cytoplasm. The fluorescent dye stains the cell nucleus with a blue stain due to its chemical affinity with the A-T region of DNA, and the Cu-BTC@PDA NWs are tagged by the green fluorescence of the FITC complex [51]. It is important to note that Cu-BTC@PDA NWs had the highest thermal response during PTT, they also lowered the ROS created during treatment to exclude cancer cell death owing to oxidative damage. Due to larger nanoparticles, we can improve the temperature distribution by increasing the distance between particles or changing the position of illumination [52].

Furthermore, the PTT effect in the Cu-BTC@PDA NWs with NIR irradiation led to concentration-dependent cell death. The cell viability decreased to 42% when the concentration of the NWs dispersions reached a high concentration (100 µg mL^−1^), suggesting that the heat produced by the NIR laser could efficiently destroy cancer cells [53]. These data clearly showed that Cu-BTC@PDA NWs were more efficient as a photothermal agent in the in vitro photothermal ablation of cancer cells.

## 5. Conclusions

Cu-BTC NWs were produced using a single-pot hydrothermal technique. After PDA coating, the PTT characteristics, MRI analysis, and in vitro PTT therapeutic effects of the NWs were evaluated.

- The Cu-BTC@PDA NWs exhibited good PTT conversion efficiency and thermal stability.
- The Cu-BTC@PDA NWs exhibited an excellent T_1_ MRI contrast agent.
- The results in vitro demonstrated the efficacy of PTT therapeutic effects and their biocompatibility.
- Cu-BTC@PDA NWs effectively scavenged the large amount of ROS generated during PPT, thus improving the toxic side effects caused by these ROS on healthy tissues.

Therefore, NIR light-responsive Cu-BTC@PDA NWs were used as potential theranostic agents that can be employed for MRI and photothermal ablation in vitro. In addition, this study may encourage further examination of multifunctional MOFs for synergistic cancer theranostics. Moreover, the results of our investigation can be used to build PTT agents for the safe eradication of the fatal disease.

### Future Perspectives

Although we have seen good biocompatibility and Fenton/Fenton-like reactions of Cu ions in Cu-BTC@PDA NWs, we are going to combine SERS and chemodynamic therapy with photothermal therapy, to promote their theranostic applications for cell tracking and synergistic chemodynamic/photothermal therapy of tumors in vitro and in vivo.

## Data Availability

The data presented in this study are available upon request from the corresponding author.
